# Secondary Localized Cutaneous Amyloidosis is not Rare in Bowen’s Disease and Bowenoid Papulosis

**DOI:** 10.5146/tjpath.2021.01530

**Published:** 2022-01-21

**Authors:** Can Baykal, Ozge Hurdogan, Goncagul Babuna Kobaner, Algun Polat Ekinci, Nesimi Buyukbabani

**Affiliations:** Department of Dermatology & Venereology, Istanbul University, Istanbul Faculty of Medicine, Istanbul, Turkey; Department of Pathology, Istanbul University, Istanbul Faculty of Medicine, Istanbul, Turkey

**Keywords:** Bowen’s disease, Cutaneous amyloidosis, Regression, Bowenoid papulosis

## Abstract

Secondary localized cutaneous amyloidosis is a histopathological finding seen in the dermis, in various benign, premalignant, and malignant skin conditions, without clinical significance. The real incidence is not known. We aimed to investigate the phenomenon of secondary localized cutaneous amyloidosis in Bowen’s disease and Bowenoid papulosis. We retrospectively evaluated the data of all cases with histopathological confirmation of Bowen’s disease and Bowenoid papulosis between 2006 and 2017 in our Dermatovenereology and/or Pathology departments. Secondary localized cutaneous amyloidosis was observed in three patients with Bowen’s disease (3/52; 5.8%) and in three patients with Bowenoid papulosis (3/18; 16.7%). Herein, we present the demographic, clinical and histopathological features of these six cases of secondary localized cutaneous amyloidosis in detail. Although the occurrence of secondary localized cutaneous amyloidosis in epithelial tumors is a well-known phenomenon, its incidence has not been previously reported in Bowen’s disease and Bowenoid papulosis. Therefore, our results indicating a high incidence may be particularly important for Bowenoid papulosis, as its association with secondary localized cutaneous amyloidosis has only been shown in one case before. Moreover, in three of six cases, we histologically observed areas of regression with a marked prominence of amyloid deposition. Remarkably, two of these patients had a history of topical application of destructive agents which reveals a possible etiologic relationship between secondary localized cutaneous amyloidosis and cellular apoptosis/necrosis induced by these external agents.

## INTRODUCTION

Besides the involvement of the skin in primary systemic amyloidosis, amyloid deposition may be seen in primary localized cutaneous amyloidosis (PLCA) causing specific macular or papular skin lesions, and in various benign and malignant skin conditions as a “secondary” histopathological finding without clinical significance. In cases with secondary localized cutaneous amyloidosis (SLCA), the amount of amyloid deposition is usually scant and hard to visualize, and thus susceptible to be overlooked in routine hematoxylin and eosin (H&E) stained sections (1).

In our practice over the past 11 years, we observed SLCA in tissue specimens of six patients with Bowen’s disease (BD) or Bowenoid papulosis (BP), raising the question whether this association may be more common than estimated. Moreover, in three of these cases (two with BD and one with BP), we histologically observed areas of regression in which amyloid deposition in the papillary dermis paralleled partial regression of the lesion. As two of these patients had a history of topical application of destructive agents (silver nitrate stick and quicklime based ointment), a possible etiologic relationship between SLCA and cellular apoptosis/necrosis induced by these agents is suggested.

## CASE REPORTS

We retrospectively evaluated the data of 70 consecutive patients who were diagnosed with BD (52 patients) or BP (18 patients) on the basis of typical clinical and histopathological features between 2006 and 2017 at the Dermatovenereology and/or Pathology departments. All tissue specimens were examined by the same experienced dermatopathologist. Secondary localized amyloid deposition was observed in a total of six patients, three with BD and three with BP.

**Figure 1:**
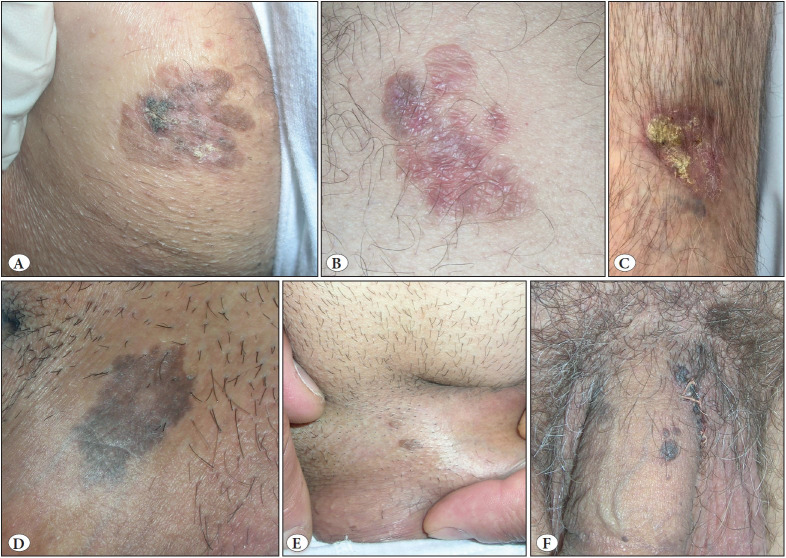
**A)** (Case 1) A 3 cm-sized, irregular pigmented, slightly hyperkeratotic solitary plaque of Bowen’s disease on left groin. **B)** (Case 2) A 2-cm sized, erythematous slightly raised solitary flat plaque of Bowen’s disease, showing foci of regression on posterior aspect of right leg. **C)** (Case 3) A 2-cm sized plaque of Bowen’s disease with development of superficially invasive squamous cell carcinoma on the left side of the lesion as a crusted area on the anterior aspect of left lower leg. **D)** (Case 4) A hyperpigmented flat plaque of bowenoid papulosis on penile shaft. **E)** (Case 4) Two small hyperpigmented papules of bowenoid papulosis, developed after the excision of the initial plaque lesion. **F)** (Case 5) Multiple clustered hyperpigmented dome-shaped papules of bowenoid papulosis on penile shaft.

**Figure 2:**
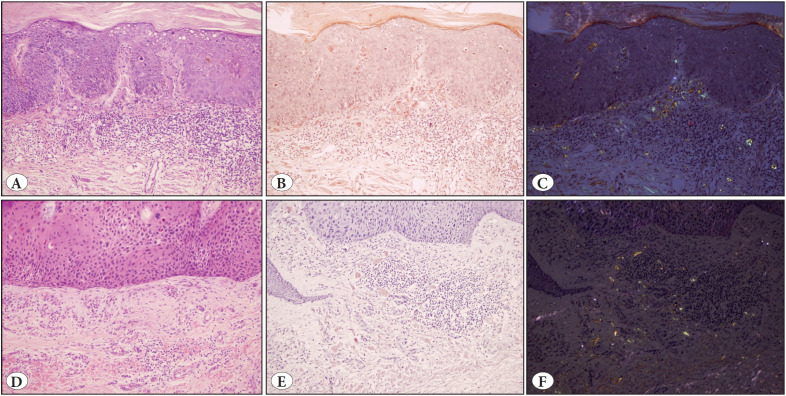
Bowen’s disease: **A)** (Case 1) Parakeratosis, acanthosis and full thickness keratinocyte atypia in the epidermis, increased mitosis, diskeratosis, eosinophilic homogeneous globular material deposition and mononuclear inflammatory infiltrate in the papillary dermis (H&E; x200). **B)** (Case 1) Congophilic droplets in the papillary dermis (Congo red; x200). **C)** (Case 1) Droplets showing birefringence under polarized light (Congo red-polarized light; x200). **D)** (Case 3) Area of regression showing epidermal keratinocyte atypia and homogeneous eosinophilic material deposition in papillary dermis (H&E; x200). **E)** (Case 3) Congophilic droplets in the papillary dermis (Congo red; x200). **F)** (Case 3) Deposits showing birefringence (Congo red-polarized light; x200).

**Figure 3:**
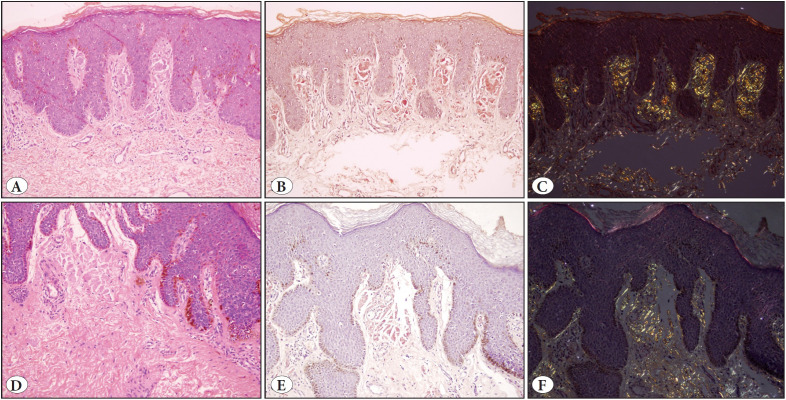
Bowenoid papulosis: **A)** (Case 4) Bowenoid histopathology and eosinophilic substance accumulation filling the dermal papillae (H&E; x200). **B)** (Case 4) Congophilic material accumulation in the papillary dermis (Congo red; x200). **C)** (Case 4) Deposits showing birefringence (Congo red-polarized light; x200). **D)** (Case 5) Atypical keratinocytes, mitoses and apoptotic cells in the epidermis and eosinophilic material in the papillary dermis (H&E; x200). **E)** (Case 5) Deposits are congophilic (Congo red; x200). **F)** (Case 5) Birefringence under polarized light (Congo red-polarized light; x200).

All patients showed classical clinical features of BD (Figure 1A-C) or BP (Figure 1D-F) and in one patient with BD, a raised nodule representing superficially invasive squamous cell carcinoma (SCC) had developed in one area of the plaque (Figure 1C). The presence of amyloid deposition was incidentally detected as an eosinophilic material in the papillary dermis during routine histopathological examination, and was further confirmed with special stains, Congo red and crystal violet. Congo red stained sections were examined under polarized light and birefringence confirmed the presence of amyloid (Figure 2B,C, 2E,F and Figure 3B,C, Figure 3E,F).

Case 1 was a 73-year-old woman who had a 3 cm-sized, irregular pigmented, slightly hyperkeratotic solitary plaque of BD on the right groin that had developed 6 months ago (Figure 1A). A punch biopsy showed hyperkeratosis, lack of maturation, loss of polarity in all epidermal layers, and apoptotic keratinocytes, consistent with BD. There were congophilic droplets in the papillary dermis (Figure 2B).

Case 2, a 58-year-old man, had a 3-year history of an erythematous slightly raised, solitary flat plaque of BD with a diameter of approximately 2 cm on the posterior aspect of the right leg (Figure 1B). There were skin-colored flat areas suggesting regression. The patient mentioned application of silver nitrate stick to the lesion, upon the recommendation of a friend, approximately one month ago. A punch biopsy showed epidermal keratinocyte atypia consistent with BD and the lesion was completely excised. Histopathological examination of the excision revealed typical BD showing also an area of regression with a slightly atrophic epidermis lacking findings reminiscent of BD. Papillary dermis was full of homogeneous eosinophilic material deposition, later proven to be amyloid.

Case 3 was a 76-year-old man presented with a 2 cm-sized plaque of BD associated with the development of superficially invasive squamous cell carcinoma on the anterior aspect of the left lower leg. The lesion was slightly raised and crusted on the left side (Figure 1C). The patient mentioned that his lesion first appeared 40 years ago and that recently upon recommendation of a friend, he applied quicklime base ointment. Last application was 15 days prior to his admission. A punch biopsy revealed epidermal keratinocyte atypia consistent with BD and the lesion was completely excised. In addition to the typical features of BD and invasive carcinoma, a sample taken from the area of regression showed epidermal keratinocyte atypia in a small focus and amyloid accumulation in the papillary dermis as aggregates of eosinophilic droplets (Figure 2D-F).

Case 4, a 64-year-old man, had a 2-year history of hyperpigmented flat plaque of BP (Figure 1D) which was accompanied by pigmented papules (Figure 1E) on the penile shaft. A punch biopsy from the plaque revealed Bowenoid epidermal histopathology and eosinophilic substance accumulation in the papillary dermis, showing birefringence under polarized light (Figure 3A-C).

Case 5 was a 66-year-old man who had experienced multiple, clustered hyperpigmented dome-shaped papules of BP on the penile shaft (Figure 1F) for the last 3 years. An excisional biopsy of a papular lesion revealed few dispersed atypical keratinocytes. Mitoses and apoptotic cells were visible with a prominent basal layer pigmentation. Eosinophilic substance accumulation filling the dermal papilla was also observed (Figure 3D-F).

Case 6, a 59-year-old man, presented with two hyperpigmented flat plaques of BP on the pubic and scrotal areas which appeared 27 years ago, and showed a persistent course. A punch biopsy revealed Bowenoid epidermal histopathology with keratinocyte atypia associated with hyperkeratosis. The papillary dermis contained aggregates of amyloid. Edema, scattered melanophages and band-like lymphocytic infiltration, more intense in the lesional area, were highly suggestive of regression. However, the patient could not clearly remember whether he had applied a topical destructive agent to the lesion.

The demographic, clinical and histopathological features of these six cases with SLCA are summarized in Table 1.

**Table 1:** The demographic, clinical and histopathological features of the patients with Bowen’s disease and Bowenoid papulosis associated with secondary localized cutaneous amyloidosis.

**Patient**	**1**	**2**	**3**	**4**	**5**	**6**
**Age (years) / Gender**	73 / F	58 /M	76 / M	64 / M	66 / M	59 / M
**Duration of the lesions**	6 months	3 years	40 years	2 years	3 years	27 years
**Location of the lesions**	Groin	Right leg	Left lower leg	Penile shaft	Penile shaft	Pubis, scrotum
**Size of the lesions**	3 cm	2 cm	2 cm	1.5 cm, 0.4 cm	0.3-0.5 cm	2 cm, 3 cm
**Clinical morphology**	Solitary hyperkeratotic plaque	Solitary erythematous flat plaque	Solitary plaque with crusted ulceration	A hyperpigmented flat plaque and a few papules	Multiple hyperpigmented dome-shaped papules	Two hyperpigmented plaques with irregular surface
**History of exposure to destructive agents**	(-)	(+) Silver nitrate stick	(+) Quicklime based ointment	(-)	(-)	(-)
**Histopathological** **features (H&E)**	Full thickness keratinocyte atypia in the epidermis, eosinophilic substance deposition	Full thickness keratinocyte atypia in the epidermis, eosinophilic substance deposition	Superficially invasive squamous cell carcinoma developed on BD, eosinophilic substance deposition	Full thickness keratinocyte atypia in the epidermis, eosinophilic substance deposition	Dyskeratosis, eosinophilic substance deposition	Hyperkeratosis, acanthosis, keratinocytes with giant nuclei in basal and suprabasal layers, apoptotic cells, eosinophilic substance deposition
**Congo red** **Crystal violet**	(+) (+)	NA (+)	(+) (+)	(+) (+)	(+) (+)	(+) (+)
**Diagnosis**	BD + SLCA	BD + SLCA	BD +SCC +SLCA	BP + SLCA	BP + SLCA	BP + SLCA

**H&E:** Hematoxylin and eosin, **BD:** Bowen’s Disease, **SLCA:** Secondary localized cutaneous amyloidosis, **SCC:** Squamous cell carcinoma, **BP: **Bowenoid papulosis.

## DISCUSSION

SLCA is most commonly associated with skin tumors of epithelial origin such as seborrheic keratosis, actinic keratosis, BD, basal cell carcinoma (BCC), and SCC (1). In these epithelial tumors, amyloid deposition is restricted to the dermo-epidermal junction / papillary dermis or to the tumoral stroma, similar to our observations in our six cases with BD and BP (1,2).

The mechanism of amyloid deposition in PLCA and SLCA has been postulated to be similar. Literature findings suggest that amyloid is derived from degenerated epidermal cells and keratin released from apoptotic basal keratinocytes or colloid bodies is the precursor protein (3). Keratin tonofilaments of epidermal keratinocytes undergo “filamentous degeneration” resulting in amyloid formation. Moreover, two β-sheet-rich proteins, galectin-7 and actin, were recently suggested to be the components of amyloid deposition in localized cutaneous amyloidosis (2).

SLCA may also be associated with local, chronic inflammatory conditions that may also induce apoptotic processes (4,5). It is hypothesized that “drop off” of epidermal keratinocytes into the dermis and the interaction between epidermal components and fibroblasts are essential for the formation of amyloid precursors (3). In accordance with current hypothesis, Cases 2 and 3 with BD, who used topical agents with tissue destructive effects (silver nitrate and quicklime based ointment, respectively), showed conspicuous regression as well as marked prominence of amyloid deposition in the areas of regression. To the best of our knowledge, amyloid deposition in the papillary dermis which paralleled areas of regression, probably induced by application of tissue destructive agents, has not previously been reported in BD and BP. A similar regression associated with amyloid deposition observed in one of our BP patients (Case 6) comforts the same hypothesis. In this patient, the reason for regression could not be identified.

There is a paucity of studies investigating the incidence of amyloid deposition in epithelial tumors. In one series of 199 BCCs, the frequency of amyloid deposition was found to be only 8% in H&E stained sections, but increased to 51% using special stains (6). In these series, the solid, adenoid and cystic types of BCC were found to be more commonly associated with SLCA (6). Additionally, a rarer association without a clinical significance of SLCA with other skin conditions like disseminated superficial actinic porokeratosis, melanocytic nevi, mycosis fungoides, PUVA-exposed skin, discoid lupus erythematosus, and dermatofibroma has also been reported (1,4,5).

BD, an in-situ SCC, presents typically with a slow-growing erythematous patch or plaque of a few centimeters. Cumulative risk for invasive SCC within 5 years of a diagnosis of BD has been reported to be 11.7% and 6.9% in men and women, respectively (7). Although the association of SLCA with BD is considered to be a well-known phenomenon, literature data regarding this association is only restricted to a few cases or small case series (1,8). Furthermore, no secondary amyloid deposition was observed in a series of six cases with BD (9).

BP is an uncommon sexually transmitted disease induced by human papilloma virus infection. BP manifests as multiple or solitary, asymptomatic, brownish-violaceous pigmented dome-shaped papules or flat plaques in the anogenital area, with a variable course. Clinically, the most common differential diagnosis includes condyloma acuminata, whereas histopathological features resemble SCC in-situ, namely BD. Therefore, the distinction between BP and BD is usually based on clinical findings, especially location. In BP, association with SLCA has only been reported in one case so far (10). Remarkably, in our large series with BD and BP, the incidence of SLCA was found to be 5.8% in BD and 16.7% in BP. Furthermore, the real incidence of SLCA association may be higher in both diseases as amyloid stains are not routinely performed in all cases.

In conclusion, our results may be particularly important for BP, as its association with SLCA is not well-known and thus maybe an under-reported phenomenon in the literature. As all of our patients with SLCA showed clinical features typical for BD or BP, this rare association seems to be clinically insignificant. Moreover, amyloid deposition in the papillary dermis showing a marked prominence in the areas of regression reveals a possible etiologic relationship between SLCA and therapeutic or destructive agents inducing cellular apoptosis/necrosis which has not previously been reported in the literature.

## Conflict of Interest

The authors declare no conflict of interest.
